# Effects of hydrogen sulfide on inflammation in caerulein-induced acute pancreatitis

**DOI:** 10.1186/1476-9255-6-35

**Published:** 2009-12-30

**Authors:** Jenab N Sidhapuriwala, Siaw Wei Ng, Madhav Bhatia

**Affiliations:** 1 Cardiovascular Biology Research Group, Department of Pharmacology, Yong Loo Lin School of Medicine, CRC MD11, National University of Singapore 117597, Singapore, Singapore; 2 University of Oxford Department of Physiology, Anatomy and Genetics Sherrington Building, Parks Road, Oxford OX1 3PT, UK

## Abstract

**Background:**

Hydrogen sulfide (H_2_S), a gaseous mediator plays an important role in a wide range of physiological and pathological processes. H_2_S has been extensively studied for its various roles in cardiovascular and neurological disorders. However, the role of H_2_S in inflammation is still controversial. The current study was aimed to investigate the therapeutic potential of sodium hydrosulfide (NaHS), an H_2_S donor in *in vivo *model of acute pancreatitis in mice.

**Methods:**

Acute pancreatitis was induced in mice by hourly caerulein injections (50 μg/kg) for 10 hours. Mice were treated with different dosages of NaHS (5 mg/kg, 10 mg/kg or 15 mg/kg) or with vehicle, distilled water (DW). NaHS or DW was administered 1 h before induction of pancreatitis. Mice were sacrificed 1 h after the last caerulein injection. Blood, pancreas and lung tissues were collected and were processed to measure the plasma amylase, myeloperoxidase (MPO) activities in pancreas and lung and chemokines and adhesion molecules in pancreas and lung.

**Results:**

It was revealed that significant reduction of inflammation, both in pancreas and lung was associated with NaHS 10 mg/kg. Further the anti-inflammatory effects of NaHS 10 mg/kg were associated with reduction of pancreatic and pulmonary inflammatory chemokines and adhesion molecules. NaHS 5 mg/kg did not cause significant improvement on inflammation in pancreas and associated lung injury and NaHS 15 mg/kg did not further enhance the beneficial effects seen with NaHS 10 mg/kg.

**Conclusion:**

In conclusion, these data provide evidence for anti-inflammatory effects of H_2_S based on its dosage used.

## Background

Hydrogen sulphide (H_2_S) a novel gaseous messenger, is synthesized endogenously from L-cysteine by two pyridoxal-5'-phosphate-dependent enzymes, cystathionine β-synthetase (CBS, EC4.2.1.22) and cystathionine γ-lyase (CSE, EC4.4.1.1). Both CBS and CSE are widely distributed in tissues. However, CBS is the predominant source of H_2_S in the central nervous system whereas CSE is the major H_2_S-producing enzyme in the cardiovascular system. H_2_S dilates blood vessels and relaxes gastrointestinal smooth muscles by opening muscle K_ATP _channels and promotes hippocampal long-term potentiation by enhancing the sensitivity of N-methyl-D-aspartate receptors to glutamate [1,2].

Since the discovery of endogenous H_2_S, many studies have been performed to understand the physiologic and pathologic roles of this gas and numerous animal studies have shown its beneficial effects especially in cardiovascular disorders [2]. However the role of H_2_S in inflammation is only recently beginning to emerge and the exact role of H_2_S in inflammation is still not very clearly understood. Research studies have shown pro-inflammatory effects of H_2_S in various models of inflammation. In those models of inflammation plasma H_2_S level, tissue H_2_S synthesizing enzyme activity and CSE expression were increased and inhibition of H_2_S synthesis by DL-propargylglycine (PAG) treatments reduced the inflammation [3-8]. In addition, some studies have also reported anti-inflammatory effects of H_2_S. Treatments with either H_2_S releasing non steroidal anti-inflammatory drugs (e.g. s-diclofenac, ATB-429) or with H_2_S donors (e.g. sodium hydrosulfide, Lawesson's reagent or N-acetylcysteine) have demonstrated anti-inflammatory activity in various models of inflammation [9-15]. Recent studies have also shown biphasic dose response of H_2_S in inflammation. In myocardial ischemia reperfusion injury, treatment with different doses of H_2_S ranging from (10-500 μg/kg) revealed U shaped dose response curve. In this study, significant reduction of infarct size was observed in mice received 50 μg/kg [16]. In another study of myocardial ischemia reperfusion injury, similar effect of H_2_S was observed. Post conditioning with exogenous sodium hydrosulfide (NaHS) treatment (0.1 to 10 μM) produced a concentration-dependent limitation of infarct. However, NaHS (100 μM) did not decrease the infarct size [17].

In the present study we investigated therapeutic potential of sodium hydrosulfide (NaHS), an H_2_S donor in *in vivo *model of acute pancreatitis in mice.

## Methods

### Experimental procedures

All animal experiments were approved by the Animal Ethic Committee of National University of Singapore and were carried out in accordance with established International Guiding Principles for Animal Research). Swiss mice (male, 20-25 g) were used and maintained in the Animal Housing Unit in an environment with controlled temperature (21-24°C) and lighting (12:12 h light-darkness cycle). Standard laboratory chow and drinking water were provided *ad libitum*. A period of at least 2 days was allowed for the animals to acclimatize before any experimental procedures were undertaken.

### Induction of acute pancreatitis

Caerulein was obtained from Bachem (Bubendorf, Switzerland) and NaHS was obtained from Sigma-Aldrich (USA). Mice were randomly assigned to control or experimental groups using 10 animals for each group. Animals were given hourly intraperitoneal (i.p.) injections of normal saline (saline control group) or saline containing caerulein (50 μg/kg) over 10 hours [4,10]. Groups of animal were treated either with different doses of NaHS (5 mg/kg, 10 mg/kg or 15 mg/kg) dissolved in distilled water (DW), or with only DW (vehicle). NaHS or DW was given i.p. one hour before the first caerulein injection. One hour after the last caerulein injection animals were sacrificed by an i.p. injection of a lethal dose of pentobarbital (50 mg/kg: Nembutal, CEVA Sante Animale, Naaldwijk, Netherlands). Blood, pancreas and lung tissues were collected. Harvested heparinized blood was centrifuged (10,000 rpm, 10 min, 4°C) and the plasma was aspirated and stored at -80°C for subsequent detection of plasma amylase. Samples of pancreas and lung were weighed, snap frozen in liquid nitrogen and then stored at -80°C for subsequent measurement of tissue myeloperoxidase (MPO) activities, chemokines and adhesion molecules as described in detail below. Parts of the pancreas and lung were also fixed in 10% vol/vol neutral phosphate-buffered formalin for more than 48 h and then were processed for histology.

### Amylase estimation

Plasma amylase activity was measured using a kinetic spectrophotometric assay. Plasma samples were incubated with the Amylase reagent (Sigma, St. Louis, Mo) for 2 min at 37°C, and absorbance was measured every minute for the subsequent 2 min at 405 nm using manufacturers' instructions [4,10]. The resulting change in absorbance was used to calculate the amylase activity.

### MPO estimation

Inflammatory cells sequestration in pancreas and lung were quantified by measuring tissue MPO activity [4,10]. Tissue samples were thawed, homogenized in 20 mM phosphate buffer (pH 7.4), centrifuged (13,000 rpm, 10 min, 4-C), and the resulting pellet resuspended in 50 mM phosphate buffer (pH 6.0) containing 0.5% wt/vol hexadecyltrimethylammonium bromide (Sigma). The suspension was subjected to four cycles of freezing and thawing and further disrupted by sonication (40 s). The sample was then centrifuged (13,000 rpm, 5 min, 4-C), and the supernatant was used for the MPO assay. The sample was mixed with equal volume of 1-component tetramethylbenzidine (TMB) substrate (Sureblue), incubated for a fixed time, and then terminated by equal volume of 2N H_2_SO4. The absorbance was measured at 450 nm and corrected for the calculated DNA [18] of the tissue sample. Results were expressed as enzyme activity (fold increase over corresponding saline injected control groups).

### Morphological examination

Paraffin-embedded pancreas and lung samples were sectioned (5 μm), stained with hematoxylin/eosin (H and E) and were examined with light microscopy.

### Enzyme-linked immunosorbent assay (ELISA) analysis of chemokines and adhesion molecules

The levels of chemokines (CCL2, CCL3 and CXCL1) and adhesion molecules (E- and P-selectins, ICAM-1, and VCAM-1) were measured in pancreas and lung tissue homogenate by a sandwich ELISA using DuoSet ELISA kits. Briefly, an anti-chemokine//adhesion molecule primary antibodies were coated onto 96- well ELISA plates and incubated overnight at room temperature. Samples and standards were added to the wells and incubated for 2 h, the wells were washed, and a biotinylated goat anti-mouse chemokine/adhesion molecule antibodies were added for 2 h. Plates were washed again, and streptavidin antibodies conjugated to HRP were added for 20 min. After a further wash, TMB was added for color development, and the reaction was terminated with 2 N H_2_SO4. Absorbance was measured at 450 nm. Sample concentration was estimated from the standard curve. The sample concentration was then corrected for the DNA content of the tissue [18].

### Statistical analysis

All values were expressed as mean ± S.E.M. The significance of changes was evaluated by using ANOVA when comparing three or more groups and Tukey and/or LSD method were used as a post hoc test for comparison among different groups. A *P *value of < 0.05 was considered to indicate a significant difference.

## Results

### Effect of different dosages of NaHS on plasma amylase in caerulein-induced acute pancreatitis

**Figure 1:**
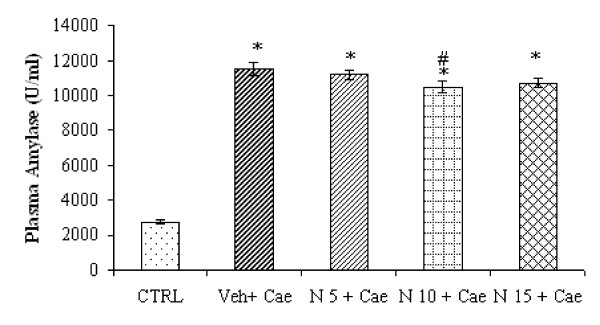
**Effect of NaHS treatment on plasma amylase activity**. Acute pancreatitis was induced by intraperitoneal administration of caerulein ((50 μg/kg, hourly for 10 h). Column labeled 'CTRL' refers to plasma amylase activity in mice injected intraperitoneal saline (not caerulein) as control. Column labeled 'Veh+Cae', 'N5+Cae', 'N10+Cae', 'N15+Cae' refers pretreatment with vehicle (DW) or different dosages of NaHS (5 mg/kg, 10 mg/kg or 15 mg/kg respectively) administered intraperitoneal 1 h before the first injection of caerulein. Results shown are the mean ± SEM for 8-10 animals in each group. Asterisk (*): P < 0.05 c.f. CTRL group. Asterisk (#): P < 0.05 c.f. (Veh + Cae) group. Abbreviations used: CTRL: Control; Cae: Caerulein; Veh: Vehicle; N: NaHS.

In our initial studies, groups of mice (n = 10) were treated with different dosages of NaHS (N5 mg/kg, N10 mg/kg and N15 mg/kg). NaHS was administered 1 h before the caerulein induced pancreatitis. Effects of NaHS were compared with the group of mice (n = 10) treated with only DW (vehicle) 1 h before the caerulein induced pancreatitis. As shown in Fig. 1, mice pretreated with vehicle or with NaHS followed by hourly caerulein injections, pancreatitis was manifested by significant rise in plasma amylase activity compared to mice injected with hourly saline only (P < 0.05). However within the NaHS group, significant reduction of plasma amylase compared to vehicle pretreated mice was not associated with mice received NaHS either 5 mg/kg or 15 mg/kg and a small but significant reduction of plasma amylase activity was observed only in mice received NaHS 10 mg/kg (P < 0.05).

### Effect of different dosages of NaHS on pancreas MPO in caerulein-induced acute pancreatitis

**Figure 2:**
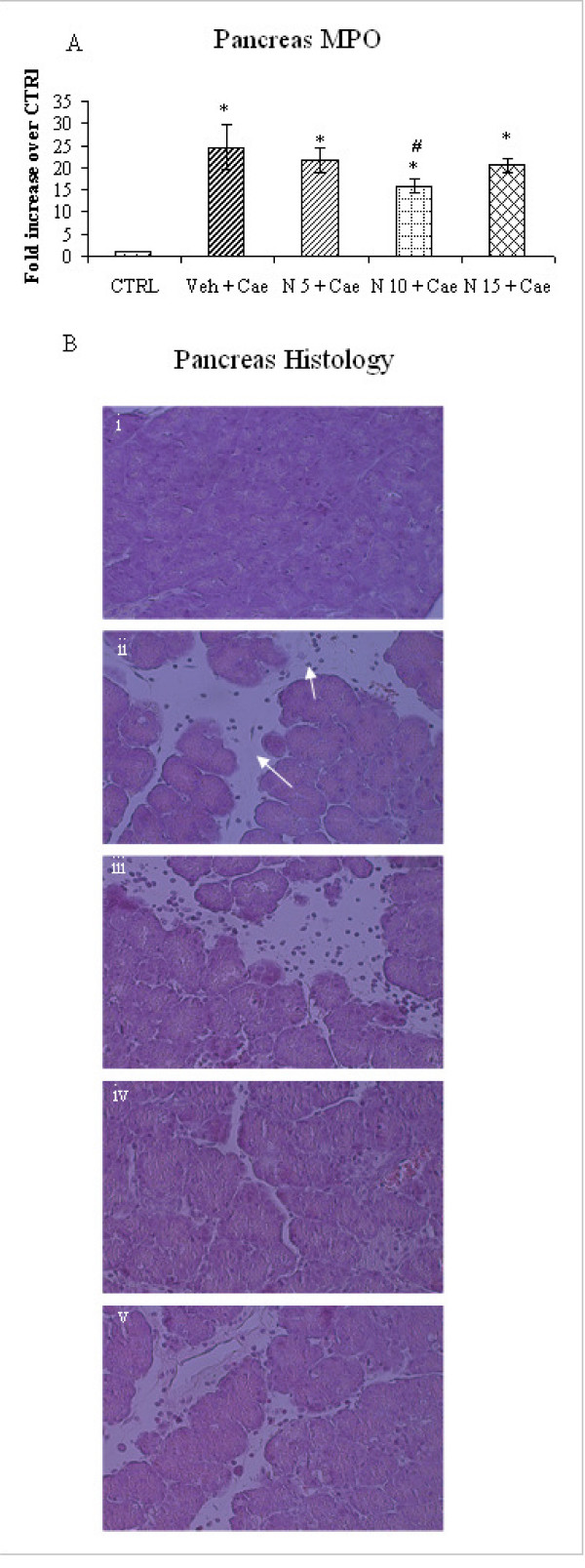
**A: Effect of NaHS treatment on pancreatic myeloperoxidase (MPO)**. Acute pancreatitis was induced by intraperitoneal administration of caerulein ((50 μg/kg hourly, for 10 h). Column labeled 'CTRL' refers to pancreas MPO activity in mice injected intraperitoneal saline (not caerulein) as control. Column labeled 'Veh+Cae', 'N5+Cae', 'N10+Cae', 'N15+Cae' refers pretreatment with vehicle or different dosages of NaHS (5 mg/kg, 10 mg/kg or 15 mg/kg respectively) administered intraperitoneal 1 h before the first injection of caerulein. Results shown are the mean ± SEM for 8-10 animals in each group. Asterisk (*): P < 0.05 c.f. CTRL group. Asterisk (#): P < 0.05 c.f. (Veh + Cae) group. Abbreviations used: CTRL: Control; Cae: Caerulein; Veh: Vehicle; N: NaHS. **B**. Pancreas histology: i, Control (saline injected) pancreas; ii, caerulein-induced pancreatitis pretreated with DW (vehicle) only; arrow showing oedema, and infiltration of inflammatory cells. iii, pretreated with NaHS (5 mg/kg);iv, pretreated with NaHS (10 mg/kg); v, pretreated with NaHS (15 mg/kg).

Further pancreatic injury was assessed by measuring pancreatic myeloperoxidase (MPO) activity and histology. Measurement of MPO enzyme which is located in azurophile granules of neutrophils and monocytes reflects inflammatory cells infiltration in tissue. There was a significant MPO increase in mice received vehicle/or various dosages of NaHS compared to saline CTRL group (Fig. 2A). However within the NaHS treated groups, only mice pretreated with NaHS 10 mg/kg had significant reduction of MPO activity as compared with vehicle treated mice (Fig. 2A). Further histological examination of pancreas sections of vehicle pre-treated mice show clear evidence of oedema, destruction of histoarchitecture of the acini and infiltration of inflammatory cells (Fig. 2B, ii). However within the NaHS groups (Fig. 2B, iii, iv and 2B, v) mice received NaHS 10 mg/kg had a significant reduction of edema and inflammatory cells compared to vehicle pretreated mice (Fig. 2B, iv).

### Effect of different dosages of NaHS on acute pancreatitis-associated lung injury

**Figure 3:**
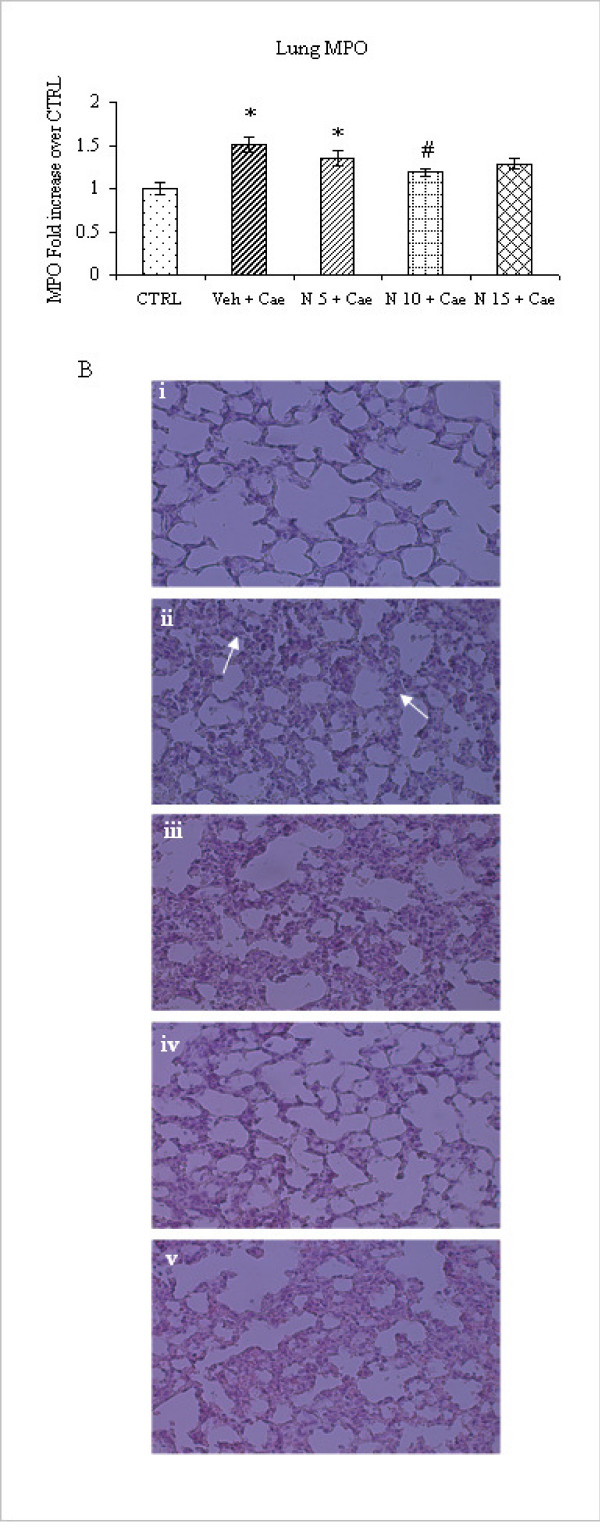
**Effect of NaHS treatment on pancreatitis associated lung injury in acute pancreatitis**. **A**. MPO activity in lung. Acute pancreatitis was induced by intraperitoneal administration of caerulein ((50 μg/kg hourly, for 10 h). Column labeled 'CTRL' refers to lung MPO activity in mice injected intraperitoneal saline (not caerulein) as control. Column labeled 'Veh+Cae', 'N5+Cae', 'N10+Cae', 'N15+Cae' refers pretreatment with vehicle or different dosages of NaHS (5 mg/kg, 10 mg/kg or 15 mg/kg respectively) administered intraperitoneal 1 h before the first injection of caerulein. Results shown are the mean ± SEM for 8-10 animals in each group. Asterisk (*): P < 0.05 c.f. control (saline) group. Asterisk (#): P < 0.05 c.f. (Veh + Cae) group. Abbreviations used: CTRL: Control; Cae: Caerulein; Veh: Vehicle; N: NaHS. **B**. Lung Histology: i, Lung section from control (saline injected) animal; ii, Lung section from caerulein-induced pancreatitis pretreated with DW (vehicle) only; arrow showing alveolar thickening and inflammatory cells infiltration. iii, pretreated with NaHS (5 mg/kg);iv, pretreated with NaHS (10 mg/kg); **v**, pretreated with NaHS (15 mg/kg).

Acute pancreatitis, in mice pretreated with DW, followed by 10 hourly injections of caerulein (50 μg/kg) was associated with lung injury. As shown in Fig. 3A caerulein-induced acute pancreatitis was associated with a significant rise in lung MPO activity, indicating the presence of sequestered inflammatory cells. Histological examination of lung sections further confirmed evidence of lung injury in acute pancreatitis as evidenced by alveolar thickening and abundance inflammatory cells infiltration (Fig. 3B, ii). However group of mice pretreated with NaHS 10 mg/kg had significant reduction of cellular infiltration as evidenced by lung MPO (Fig. 3A) and lung histology (Fig. 3B, iv), while such protection was not seen in groups of mice pretreated with NaHS 5 mg/kg or 15 mg/kg (Fig. 3A and Fig 3B, iii and v). Thus, treatment with NaHS10 mg/kg, but not with 5 mg/kg or 15 mg/kg resulted in a marked reduction in the severity of pancreatitis as well associated lung injury.

### Effect of NaHS 10 mg/kg on pancreatic and pulmonary chemokines

**Figure 4:**
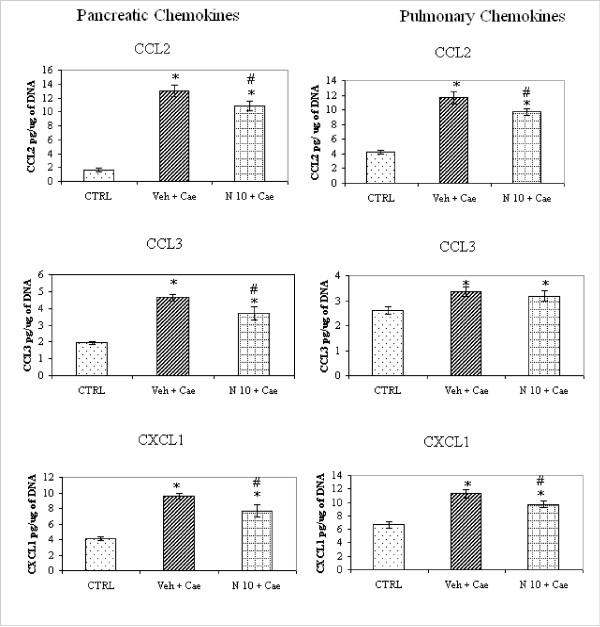
**Effect of NaHS treatment on pancreatic and pulmonary chemokines in acute pancreatitis**. Acute pancreatitis was induced by intraperitoneal administration of caerulein ((50 μg/kg hourly, for 10 h). Column labeled CTRL' refers to chemokines level in mice injected intraperitoneal saline (not caerulein) as control. Column labeled 'Veh+Cae' and 'N10+Cae' refers pretreatment with vehicle or NaHS (10 mg/kg) administered intraperitoneal 1 h before the first injection of caerulein. Results shown are the mean ± SEM for 8-10 animals in each group. Asterisk (*): P < 0.05 c.f. control (saline) group. Asterisk (#): P < 0.05 c.f. (Veh + Cae) group. Abbreviations used: CTRL: Control; Cae: Caerulein; Veh: Vehicle; N: NaHS.

Chemokines, well known for their potent leukocyte-activating properties have been shown to be involved in the pathophysiological process of experimental acute pancreatitis. Based on our initial data with different dosages of NaHS, we decided to see if reduction of pancreatic and pulmonary inflammation with NaHS 10 mg/kg has any effect on chemokines and adhesion molecules levels in pancreas and lung. As expected chemokines, Chemokine (C-C motif) Ligand 2 (CCL2), Chemokine (C-C motif) Ligand 3 (CCL3) and Chemokine (C-X-C motif) Ligand 1 (CXCL1) were significantly increased in pancreas as well as lung tissue (Fig 4: A, B and 4C) in vehicle treated group. However NaHS 10 mg/kg treatment significantly reduced all pancreatic chemokines and pulmonary chemokines except pulmonary CCL3 (Fig 4: A, B and 4C).

### Effect of NaHS 10 mg/kg on pancreatic and pulmonary cell adhesion molecules

**Figure 5:**
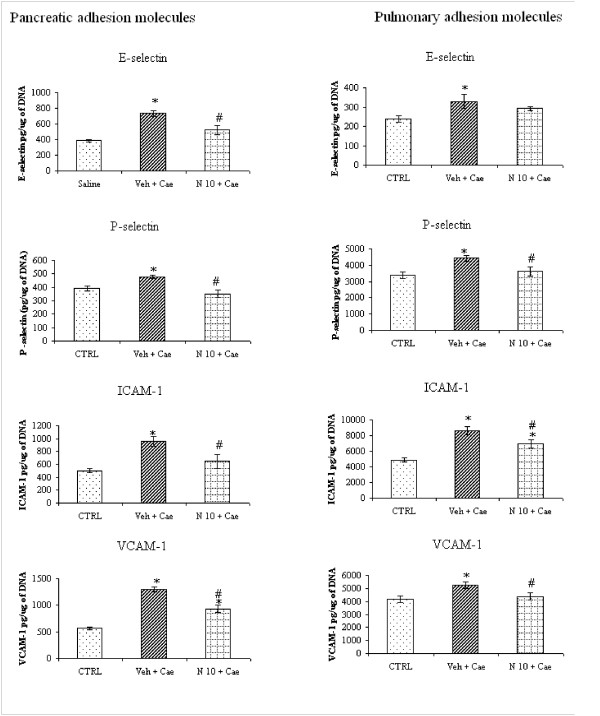
**Effect of NaHS treatment on pancreatic and pulmonary adhesion molecules**. Acute pancreatitis was induced by intraperitoneal administration of caerulein ((50 μg/kg hourly, for 10 h). Column labeled 'CTRL' refers to adhesion molecules level in mice injected intraperitoneal saline (not caerulein) as control. Column labeled 'Veh+Cae' and 'N10+Cae' refers pretreatment with vehicle or NaHS (10 mg/kg) administered intraperitoneal 1 h before the first injection of caerulein. Results shown are the mean ± SEM for 8-10 animals in each group. Asterisk (*): P < 0.05 c.f. control (saline) group. Asterisk (#): P < 0.05 c.f. (Veh + Cae) group. Abbreviations used: CTRL: Control; Cae: Caerulein; Veh: Vehicle; N: NaHS.

Pancreatic and pulmonary cell adhesion molecules E-selectin (endothelial), P-selectin (platelet), Intercellular Cell Adhesion Molecule -1 (ICAM-1) and Vascular Cell Adhesion Molecule-1 (VCAM-1) were measured by ELISA. They were significantly increased in both pancreas and lung tissue (Fig 5: A, B, C and 5D) of mice pretreated with vehicle, while NaHS 10 mg/kg significantly reduced all pancreatic and pulmonary adhesion molecules except pulmonary E-selectin (Fig 5: A, B, C and 5D).

## Discussion

Hydrogen sulfide, like nitric oxide (NO) and carbon monoxide (CO) is a biological active gas of interest to pharmacologist. Several recent publications have shown its physiological/pathological contribution mainly in cardiovascular system (CVS) and central nervous system (CNS) and its therapeutic potential in CVS and CNS disorders [1,2]. However, its precise role and therapeutic application in inflammatory disorders is still controversial. Exogenous administrations of H_2_S have shown either pro-inflammatory or anti-inflammatory effects depending on its formula, dose and disease model [5,6,9-15]. Recent studies have also shown that exogenous NaHS administration exerted biphasic therapeutic response [16,17]. The present study was aimed to investigate the therapeutic potential of exogenous NaHS (H_2_S donor) on caerulein-induced acute pancreatitis. In our initial experiment when the mice were treated with different dosages of NaHS (5 mg/kg, 10 mg/kg and 15 mg/kg) 1 h before caerulein-induced acute pancreatitis, it was revealed that there was a dose dependent reduction of plasma amylase (Fig. 1), pancreatic inflammation as evidenced by pancreas MPO and histology (Fig. 2A and 2B) and pulmonary inflammation, as evidenced by lung MPO and histology (Fig. 3A and 2B) and a significant reduction of inflammation was seen only in mice pretreated with NaHS 10 mg/kg (Fig. 1, Fig. 2A and 2B iv, Fig. 3A and 3B iv). NaHS 15 mg/kg treatment did not have any additional beneficial effect as seen with 10 mg/kg, and on contrary there is a trend towards increased inflammation as evidenced by pancreas and lung MPO and histology (Fig. 2A and 2B v, Fig. 3A and 3B v). Further in a separate experiment, mice treated with NaHS 20 mg/kg dose, was associated with increase mortality (experimental observation). Thus, there is a dose dependent effect of NaHS but doses 15 mg/kg and more are associated with toxic effects. Similar findings were also observed by other group albeit in different models. Treatment with H_2_S donor, Na_2_S in doses of (10-500 μg/kg) at the time of reperfusion and study of infarct size per area-at-risk (INF/AAR) revealed a U-shaped dose-response curve. Mice receiving 50 μg/kg displayed significant reduction in infarct size. However there is increase in ratio of INF/AAR when mice received 100 μg/kg or 500 μg/kg [16]. Similarly in another study of ischemia-reperfusion injury low physiological concentration NaHS (0.1-10 μM) reduced the infarct size in a dose-dependent manner. However high concentrate 100 μM NaHS increased the infarct size [17]. Although both these models are very different from our model, similar to our study treatment with different dosages of H_2_S donors Na_2_S or NaHS, resulted in dose dependent reduction of infarct size or inflammation and further increasing dose was not beneficial at all. The narrow therapeutic window seen with our results could be due to sudden release of H_2_S from H_2_S donor like NaHS. NaHS is water soluble, resulting in instant release of H_2_S upon injection and causing its toxic effects.

Recruitment of various inflammatory cells like neutrophils, monocytes and macrophages to the inflamed/injured tissues is mediated by chemokines. Chemokines are a group of low-molecular-weight (8-10 kDa) polypeptides and are the key components of immune surveillance [19]. We further investigated whether reduction of inflammatory cells infiltration in pancreas and lung was associated with any changes in chemokines. We investigated CC chemokines such as CCL2 and CCL3 and CXC chemokines such as CXCL1. CCL2 and CCL3 exert strong chemo-attractant effects on monocytes, macrophages, and lymphocytes. Recent studies have suggested that CCL2 is an important inflammatory mediator during the early pathophysiological process of AP and promotes distant organ failure [20]. CXCL1 is a potent chemoattractant for polymorphonuclear neutrophils (PMN) and induces neutrophil degranulation and release of lysozyme, leading to tissue damage. We found that our treatment with NaHS 10 mg/kg was associated with significant reduction of pancreatic CCL2, CCL3 and CXCL1 as well as pulmonary CCL2 and CXCL1 (Fig. 4). However there was no change in pulmonary CCL3 with NaHS treatment (Fig. 4).

We also studied the effect of NaHS on the expression of adhesion molecules in pancreas and lung. Substantial evidence indicates that adhesion molecule expression is crucial to the development and modulation of inflammatory and immune processes. Vascular adhesion molecules are important component in leukocyte rolling, adhesion and trans-endothelial migration of inflammatory cells to the site of tissue injury [19,21,22]. ICAM-1, VCAM-1, E-selectin and P-selectin have been found to play an important pro-inflammatory role in various models of acute pancreatitis [23,24]. In present study of acute pancreatitis also, there was a significant increase of ICAM-1, VCAM-1, E-selectin and P-selectin in mice pretreated with vehicle confirming their pro-inflammatory role, while pretreatment with NaHS 10 mg/kg caused significant reduction of pancreatic ICAM-1, VCAM-1, E-selectin and P-selectin as well as pulmonary ICAM-1, VCAM-1 and P-selectin (Fig. 5). There was no change in pulmonary E-selectin level with NaHS pretreatment (Fig. 5) like pulmonary CCL3 (Fig. 4). These could be due to differential regulation of inflammatory responses mediated by NaHS in pancreas and lung.

## Conclusions

In conclusion in this study of acute pancreatitis induced by hourly caerulein administration, pretreatment by different dosages of NaHS (5 mg/kg, 10 mg/kg and 15 mg/kg) revealed that NaHS 10 mg/kg was associated with down-regulation of inflammation both in pancreas and lung and it was accompanied by reduction of pro-inflammatory chemokines and adhesion molecules. In addition, these results have further demonstrated dose dependent effects of NaHS in inflammation and thus confirm hydrogen sulfide as a novel gaseous transmitter that exerts dual effects in various pathophysiological conditions. Thus, an H_2_S-releasing compound, at low doses, may represent a potential pharmacological approach in the treatment of inflammation. A lot of research is on going to develop novel H_2_S donors and this line of research would, hopefully, provide a better solution to fight against the inflammatory disorders.

## Abbreviations

H_2_S: Hydrogen sulfide; NaHS: Sodium hydrosulfide; MPO: Myeloperoxidase; CBS: Cystathionine β-synthetase; CSE: Cystathionine γ-lyase; PAG: DL-propargylglycine; TMB: tetramethylbenzidine; ELISA: Enzyme-linked immunosorbent assay; CCL2: Chemokine (C-C motif) Ligand 2; CCL3: Chemokine (C-C motif) Ligand 3; CXCL1: Chemokine (C-X-C motif) Ligand 1; ICAM: Intercellular Cell Adhesion Molecule-1; VCAM-1: Vascular Cell Adhesion Molecule-1; NO: Nitric oxide; CO: Carbon monoxide; CVS: Cardiovascular system; CNS: Central nervous system; PMN: Polymorphonuclear neutrophils.

## Competing interests

The authors declare that they have no competing interests.

## Authors' contributions

JNS designed the study and it was approved by MB. JNS and SWN conducted animal experiments and did the plasma amylase, MPO assay, histology and ELISA. MB supervised all the experiments. JNS wrote the manuscript and MB reviewed and edited the manuscript. All authors read and approved the final manuscript.
